# New Antifungal Pyranoisoflavone from *Ficus tikoua* Bur.

**DOI:** 10.3390/ijms13067375

**Published:** 2012-06-14

**Authors:** Shaopeng Wei, Wenjun Wu, Zhiqin Ji

**Affiliations:** College of Plant Protection and Institute of Pesticide Science, Northwest A & F University, Yangling, Shaanxi 712100, China; E-Mails: weishaopeng8888@163.com (S.W.); wuwenjun@nwsuaf.edu.cn (W.W.)

**Keywords:** *Ficus tikoua* Bur., pyranoisoflavone, antifungal activity

## Abstract

Considering the undesirable attributes of synthetic fungicides and the availability of Ficus species in China, the stem of *Ficus tikoua* Bur. was investigated. One new antifungal pyranoisoflavone, 5,3′,4′-trihydroxy-2″,2″-dimethylpyrano (5″,6″:7,8) isoflavone (**1**), together with two known isoflavones, wighteone (**2**) and lupiwighteone (**3**) (with previously reported antifungal activities), were isolated from ethyl acetate extract by bioassay-guided fractionation. Their structures were determined by spectroscopic analysis, such as NMR (^1^H-^1^H COSY, HMQC, HMBC and NOESY), IR, UV and HRMS, as well as ESI-MS^n^ analyses. The antifungal activities of **1**–**3** against *Phytophthora infestans* were evaluated by direct spore germination assay, and the IC_50_ values were 262.442, 198.153 and 90.365 μg·mL^−1^, respectively.

## 1. Introduction

The plants of the Ficus genus have attracted considerable attention from pharmacologists due to a wide range of biological properties. *Ficus tikoua* Bur., a wood plant of the Ficus genus, is widely distributed in southern China, India, Vietnam and Laos. It has long been used in traditional folk medicine to treat diseases, such as chronic bronchitis, diarrhea, dysentery, rheumatism, edema, impetigo, and so on [1–3]. Previous studies show that the extracts of the plant exhibit excellent antifungal activity against many species of pathogens, such as *Pseudoperonospora cubensis*, *Phytophthora infestans* and *Plasmopara viticola* [4]. The plant can be considered as a potential botanical pesticide. However, to our best knowledge, no references about its antifungal constituents have been published.

**Figure 1:**
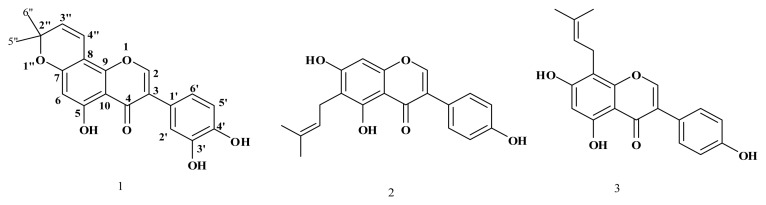
Structures of compounds **1**–**3**.

Considering the undesirable attributes of synthetic fungicides, there is an urgent need to develop alternative treatments that are less hazardous to humans and animals, and that impact less on the environment. Herein, we report the isolation, structural elucidation and antifungal activities of a new pyranoisoflavone, named 5,3′,4′-trihydroxy-2″,2″-dimethylpyrano (5″,6″:7,8) isoflavone (**1**), along with other two known isoflavones, wighteone (**2**) and lupiwighteone (**3**), whose antifungal activities were reported previously [5] (Figure 1).

## 2. Results and Discussion

### 2.1. Isolation and Structural Elucidation

The ethyl acetate-soluble fraction of the crude extract acquired for this study was successively subjected to column chromatography to yield compounds **1**–**3**. Their structures were elucidated by the methods of UV, IR, HR-ESI/MS, ESI-MS/MS and NMR.

**Figure 2:**
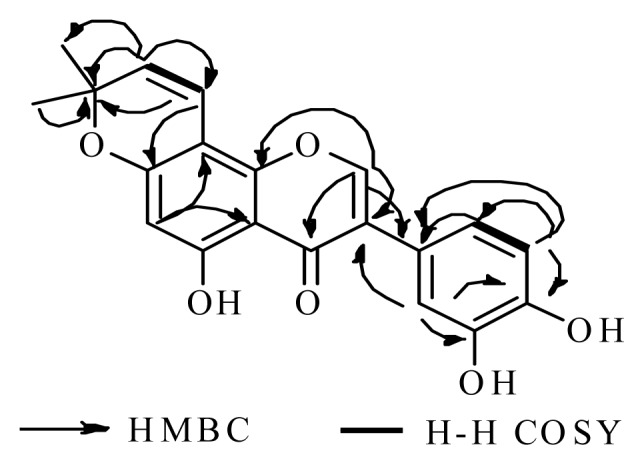
Partial structure of **1** solved by heteronuclear multiple-bond correlation (HMBC) and ^1^H-^1^H COSY spectra.

Compound **1** was obtained as a pale yellow amorphous powder. The molecular formula C_20_H_16_O_6_ was deduced from its HR-ESI-MS (observed *m/z* 353.1010 [M+H]^+^, calcd. for C_20_H_17_O_6_ 353.1020) and NMR data. IR absorption at 3410, 1686 and 1580 cm^−1^ indicated the presence of hydroxyl function, carbonyl and aromatic ring. Analysis of its NMR (Table 1) spectral data suggested the presence of the skeleton of isoflavone due to a singlet resonance at δ_H_ 8.01 and corresponding olefinic oxymethine signal at δ_C_ 156.85 are characteristic of H-2 and C-2, respectively [6–8]. The NMR spectral also revealed the presence of phenol and ketone moieties. ^13^C-NMR (DEPT) spectrum of **1** displayed 20 signals, which were assigned to two methyls, seven methines and eleven quaternary carbons (including one keto carbonyl at δ_c_ 182.8) in agreement with the molecular formula. ^1^H-NMR spectrum showed a six-proton singlet at δ_H_ 1.45 assignable to a gem-dimethyl group and two doublets (*J* = 10.1 Hz) at 6.68 and 5.71, integrating for one proton each, corresponding to vinylic protons (H-4″ and H-3″, respectively) suggesting the presence of a 2,2-dimethylchromen residue in compound **1** [9]. The signals observed at δ 6.39 (d, *J* = 2.4, 1H), δ 7.03 (d, *J* = 8.3, 1H) and δ 6.37 (dd, *J* = 2.4, 8.3, 1H) in the ^1^H-NMR of **1** were attributed to the 2′, 5′, 6′-protons, respectively. That the 2,2-dimethylchromen residue is at the 7,8-position in **1** was established based on the analysis of heteronuclear multiple-bond correlation (HMBC) spectrum and by comparing the spectral data of its monomethyl ether with that reported in literature [10,11]. The ^1^H-^1^H correlation spectroscopy (COSY) NMR spectral revealed it was possible to establish the proton sequences by the following cross-peaks: H(5′)/H(6′) and H (3″)/H(4″). Its HMBC spectrum showed many informative correlations, such as H(2)/C(3), C(4), C(9) and C(1′); H(6)/C(5), C(7), C(8) and C(10); H(2′)/C(3), C(3′) and C(4′); H(5′)/C(1′), C(4′) and C(6′); H(6′)/C(1′), C(4′) and C(5′); H(3″)/C(8) and C(2″); H(4″)/C(7) and C(2″) (Figure 2).

**Scheme 1:**
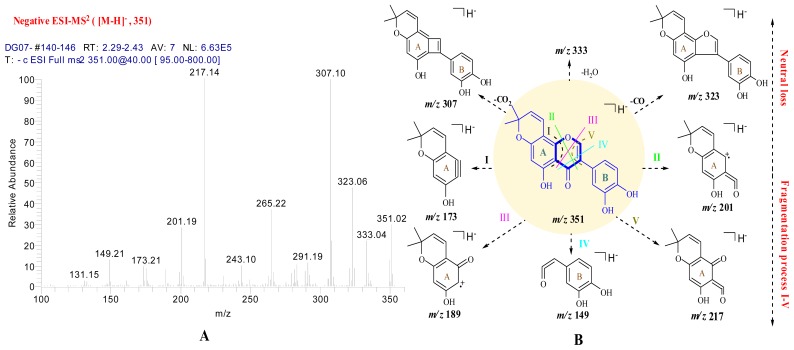
CID spectrum (**A**) and the proposed fragmentation pathways (**B**) of compound **1** in negative mode.

In order to validate the structure described above, the cleavage of compound **1** was studied by ESI-MS^n^ ether in negative mode. The major product ions of **1** observed in the tandem mass spectrometric experiments can be ascribed to five fragmentation processes according to the published literature and the five fragmentation processes, I-V, are shown in Scheme 1 [12–14]. The product ion mass spectrum of [M-H]^−^ shows two major product ions at *m*/*z* 307 and *m*/*z* 217, respectively. Fragment observed at *m*/*z* 307 might be originated from the neutral loss of CO_2_, and the *m*/*z* 217 ion has been formed in a retro-Diels Alder (RDA) reaction, that is, by fragmentation process V. Further, the product ions at *m*/*z* 173, *m*/*z* 201, *m*/*z* 189 and *m/z* 149 are the product of fragmentation processes I, II, III, IV, respectively. Finally, compound **1** was identified as 5,3′,4′-trihydroxy-2″,2″-dimethylpyrano (5″,6″:7,8) isoflavone.

Compounds **2** and **3** were two known isoflavones, wighteone (**2**) and lupiwighteone (**3**), based on UV, IR, ESI-MS, ^1^H- and ^13^C-NMR spectroscopic data [15–17].

### 2.2. Antifungal Activities

Antifungal activities of compounds **1–3** against *P. infestans* were investigated by spore germination assay, and the IC_50_ values of **1–3** were 262.442, 198.153 and 90.365 μg·mL^−1^, respectively.

As showed in Table 2, antifungal potency of compounds **2** and **3** was greater than that of compound **1**. Considering the fact that natural compounds can have synergistic antifungal activities [18], the interactions between compounds **1**–**3** need to be investigated, and we will report on this progress in the future.

## 3. Experimental Section

### 3.1. General Experimental Procedures

Melting points were measured on a WPR apparatus and are uncorrected (Shanghai Jingke Co.). IR spectra were recorded on a Nicolet FT-IR 750 spectrometer. UV spectra were obtained using a Shimadzu UV-2401A spectrometer. Optical rotations were measured with a Horiba SEPA300 polarimeter. ESI-MS/MS and HR-ESI-MS spectra were obtained on a Finnigan LCQ Advantage ion-trap mass spectrometer (Thermo Fisher Co.) and an APEX II FT-ICR mass spectrometer (Bruker Daltonics Inc.). ^1^H, ^13^C NMR, DEPT, HSQC, HMBC and NOESY spectra were obtained on Bruker Avance III-500 NMR spectrometer (Bruker Daltonics Inc.), and TMS as internal standard (^1^H at 500 MHz, ^13^C at 125 MHz, respectively).

### 3.2. Plant Material

The stem of *F. tikoua* Bur. was collected in Hongya County, Sichuan Province, P.R. China, in September 2009, and identified at the College of Life Sciences, Northwest Agricultural & Forestry University. The voucher specimens (samples No. NWAU2009-FT15) were deposited with the College of Life Sciences, Northwest Agricultural & Forestry University.

### 3.3. Extraction and Isolation

The dried and pulverized stem of *F. tikoua* Bur. (2.0 kg) was extracted three times (4 h each time) under reflux with analytical grade methanol. The whole extract was filtered and evaporated under reduced pressure at 40–45 °C using a rotary evaporator. The residue (127.0 g), equivalent to 6.35% of the weight of the dried sample, was suspended in water (2 L) and partitioned three times with ethyl acetate. The ethyl acetate fraction (31 g) was subjected to a silica gel column (500 g, 200~300 mesh, Qingdao Marine Chemical Co. Ltd., Shandong, China) and eluted with the mixture of petroleum ether-acetone-methanol of increasing polarity as eluent. Seventy fractions of *ca.* 500 mL each were collected. After analysis with thin-layer chromatography, similar fractions were combined to afford ten fractions. Then, the antifungal activities against *P. infestans* of those fractions were evaluated. The most active fraction, Fr-III (2.37 g), was injected to Sephadex LH-20 column (3.0 × 100 cm, 100 g), and eluted successively with methanol. Then, the active fractions were further purified by ODS-AP flash chromatography (50 μm, Daiso Co. Ltd., Osaka, Japan) and semi-preparative Shimadzu 6 AD HPLC apparatus (Shimadzu Co. Ltd., Tokyo, Japan) with a C_18_ preparative column (20 × 250 mm, 10 μm, flow rate 8.0 mL/min). In this way, compounds **1**–**3** were obtained.

### 3.4. Microorganism and Preparation of Zoosporangia Suspension

The tested fungal pathogen, *P. infestans* was provided by the Institute of Plant Disease, Northwest A&F University. The strain was retrieved from the storage tube and cultured for 10 days at 16 °C on rye A agar (rye infusion from 60 g·L^−1^, 20 g·L^−1^ sucrose, and 15 g·L^−1^ agar) plates. The plates were then flooded with sterile distilled water, and the zoosporangia were scraped with a glass stick. Mycelial debris was removed by filtration through double-layer cheesecloth, and the zoosporangia were harvested and suspended in sterile distilled water containing 0.1% (*v*/*v*) Tween 20. The zoosporangia were counted using a hemocytometer and adjusted to 1.0 × 10^6^ zoosporangia·L^−1^ [19,20].

### 3.5. Spore Germination (Zoospore Release from Zoosporangia) Assay

Compounds **1**–**3** (10 mg) were dissolved in dimethyl sulfoxide (DMSO, 0.1 mL) and diluted with sterile distilled water (containing 0.1% Tween 20) to prepare 10 mL stock solution, which were further diluted to prepare test solutions in which the final concentration of DMSO was <1% (*v*/*v*). A series of concentrations and one control (1% DMSO in sterile distilled water) were separately tested for zoospore release from zoosporangia of *P. infestans*. The samples were inoculated with zoosporangia suspension of *P. infestans* containing 1.0 × 10^6^ zoosporangia·L^−1^. Aliquots of 10 μL of prepared zoosporangia suspension were placed on separate glass slides in triplicate. Approximately 300 zoosporangia were observed with a light microscope after the slides containing the zoosporangia were incubated in a moist chamber at 16 °C for 6 h, and the percentages of zoospore release were calculated. Pyrimethanil (a commercial fungicide) was chosen as a positive control. Pyrimethanil inhibits the secretion of a disease protein. This fungicide exhibits potent antifungal activity against *Pseudoperonospora cubensis*, *Plasmopara viticola*, *Botrytis cinerea*, *Phytophthora infestans*, and so on [21]. The bioassay data of the antifungal activities were analyzed using SPSS 13.0 for Windows.

### 3.6. Chemistry

5,3′,4′-trihydroxy-2″,2″-dimethylpyrano(5″,6″:7,8) isoflavone (**1**): C_20_H_16_O_6_, mp 210–213 °C; UV *λ*_max_ (MeOH): 289 nm; [α]_D_ +19.0° (MeOH, *c* 1.0), IR (KBr, cm^−1^): 3410, 1686, 1580; ^1^H-NMR (MeOD, 500 MHz) δ 8.01 (1H, s, H-2), 7.03 (1H, d, *J* = 8.3 Hz, H-5′), 6.68 (1H, d, *J* = 10.1 Hz, H-4″), 6.39 (1H, d, *J* = 2.4 Hz, H-2′), 6.36 (1H, s, H-6), 6.37 (1H, dd, *J* = 2.4, 8.3 Hz, H-6′), 5.71 (1H, d, *J* = 10.1 Hz, H-3″), 1.45 (6H, s, H-5″, H-6″); ^13^C-HMR (MeOD, 125 MHz) δ 156.85 (CH-2), 122.66 (C-3), 182.84 (CO-4), 157.58 (C-5), 95.86 (CH-6), 160.90 (C-7), 106.56 (C-8), 158.87 (C-9), 106.93 (C-10), 110.63 (C-1′), 133.23 (CH-2′), 160.25 (C-3′), 157.78 (C-4′), 104.23 (CH-5′), 108.13 (CH-6′), 79.25 (C-2″), 129.67 (CH-3″), 116.09 (CH-4″) and 28.55 (2× CH_3_-5″, 6″). ESI-MS/MS: *m*/*z* (%) 351 (30) [M-H]^−^, 333 (20) [M-H-H_2_O]^−^, 323 (35) [M-H-CO]^−^, 307 (100) [M-H-CO_2_]^−^, 265 (40) [M-H-C_4_H_6_O_2_]^−^, 217 (100) [M-H-C_8_H_6_O_2_]^−^, 201 (30) [M-H-C_8_H_6_O_3_]^−^. High-resolution ESI-MS: *m*/*z* [M + H]^+^ calcd for C_20_H_17_O_6_ 353.1020; found 353.1010.

## 4. Conclusions

In summary, a new isoflavone derivative, 5,3′,4′-trihydroxy-2″,2″-dimethylpyrano (5″,6″:7,8) isoflavone (**1**), was isolated using bioactivity-guided bioassay-guided fractionation. The *in vitro* antifungal activity of **1** against *P. infestans* was preliminarily evaluated using the spore germination assay. The IC_50_ value of **1** was 262.442 μg·mL^−1^, which showed the equivalent antifungal activity as a reference fungicide, pyrimethanil with an IC_50_ value 210.050 μg·mL^−1^.

## Supplementary Information



## Figures and Tables

**Table 1:** The ^1^H and ^13^C-NMR chemical shifts of compound **1** in CD_3_OD.

Positions	δ_H_ (ppm)	δ_C_ (ppm)	HMBC
2	8.01 (s, 1H)	156.85 (CH)	C-3, C-4, C-9, C-1′
3	/	122.66 (C)	
4	/	182.84 (CO)	
5	/	157.58 (C)	
6	6.36 (s, 1H)	95.86 (CH)	C-5, C-7, C-8, C-10
7	/	160.90 (C)	
8	/	106.56 (C)	
9	/	158.87 (C)	
10	/	106.93 (C)	
1′	/	110.63 (C)	
2′	6.39 (d, *J* = 2.4, 1H)	133.23 (CH)	C-3, C-3′, C-4′
3′	/	160.25 (C)	
4′	/	157.78 (C)	
5′	7.03 (d, *J* = 8.3, 1H)	104.23 (CH)	C-1′, C-4′, C-6′
6′	6.37 (dd, *J* = 2.4, 8.3, 1H)	108.13 (CH)	C-1′, C-4′, C-5′
2″	/	79.25 (C)	
3″	5.71 (d, *J* = 10.1, 1H)	129.67 (CH)	C-8, C-2″
4″	6.68 (d, *J* = 10.1, 1H)	116.09 (CH)	C-7, C-2″
5″	1.45 (s, 3H)	28.55 (CH_3_)	
6″	1.45 (s, 3H)	28.55 (CH_3_)	

δ_C_ (ppm) 125MHz; δ_H_ (ppm) 500 MHz; multiplicities; *J* values (Hz) in parentheses. Assignments are based on DEPT and ^1^H-^13^C (HSQC and HMBC) experiments.

**Table 2:** Antifungal activities of compounds **1**–**3** against *P. infestans*.

Compounds	IC_50_ (μg·mL^−1^)	SD
1	262.442	0.16
2	198.153	0.67
3	90.365	0.45
Pyrimethanil	210.050	0.77
