# Supplementary material for: New Antifungal Pyranoisoflavone from Ficus tikoua Bur
Source: Int J Mol Sci. 2012 Jun 14;13(6):7375–82. doi: 10.3390/ijms13067375 (PMC3397532; doi:10.3390/ijms13067375)
Supplement: Supplementary file 1 [file ijms-13-07375-s001.pdf]

New Antifungal Pyranoisoflavone from *Ficus tikoua* Bur.

Supplementary Information

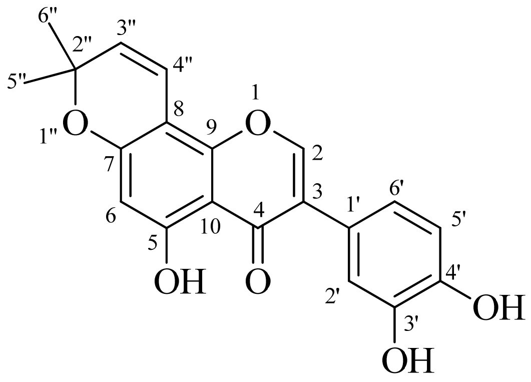

1

Figure S1. ^{1}H NMR Spectrum of **1** in CD_3OD..2
Figure S2. ^{13}C and DEPT NMR Spectrum of **1** in CD_3OD...3
Figure S3. HSQC Spectrum of **1** in CD_3OD...4
Figure S4. ^{1}H-^{1}H COSY Spectrum of **1** in CD_3OD...5
Figure S5. HMBC Spectrum of **1** in CD_3OD...6
Figure S6. NOESY Spectrum of **1** in CD_3OD..7
Figure S7. HR-ESI-MS of **1**..8

Figure S1. ^{1}H NMR Spectrum of **1** in CD_3OD.

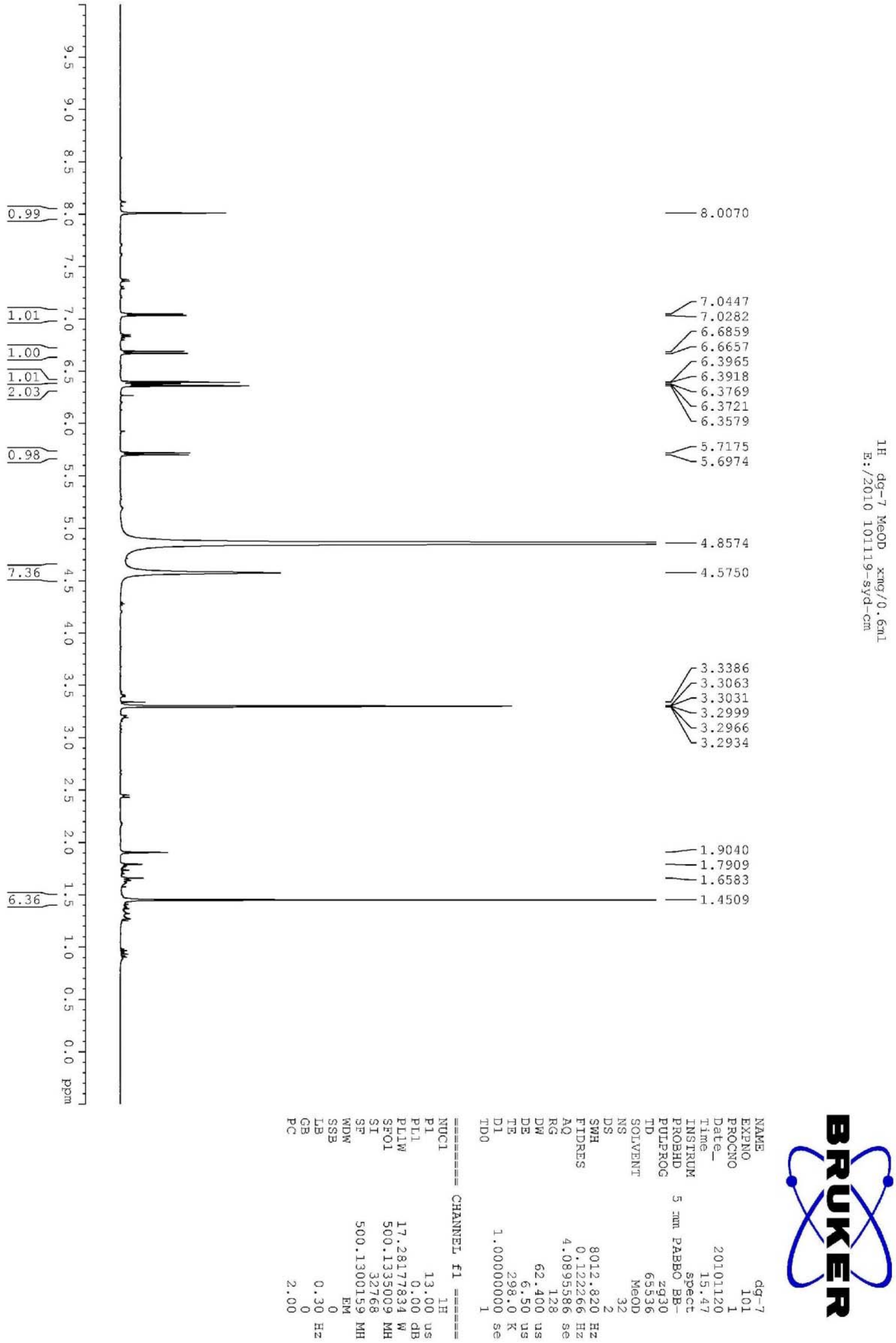

Figure S2. ^{13}C and DEPT NMR Spectrum of 1 in CD_3OD.

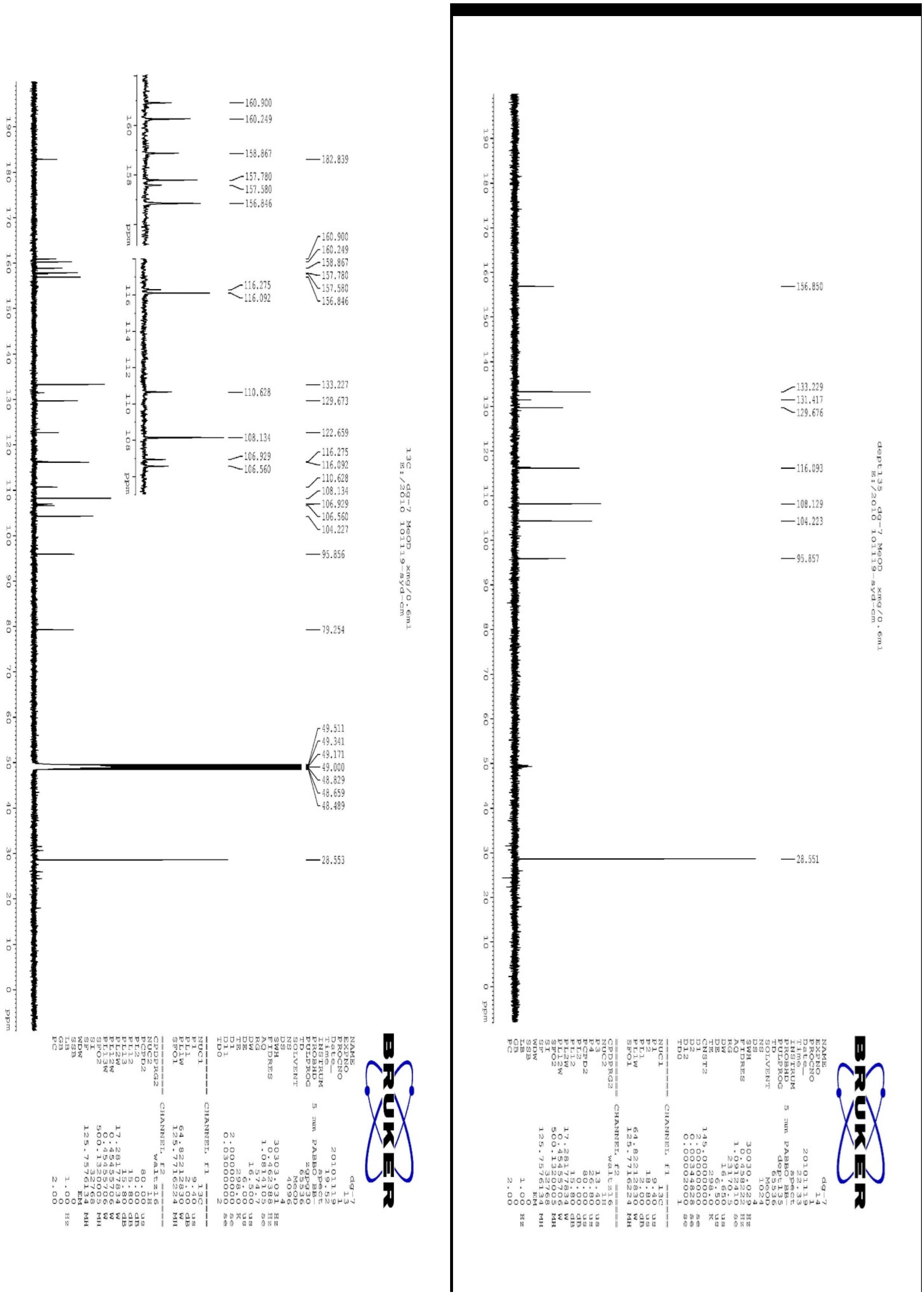

Figure S3. HSQC Spectrum of **1** in CD_3OD.

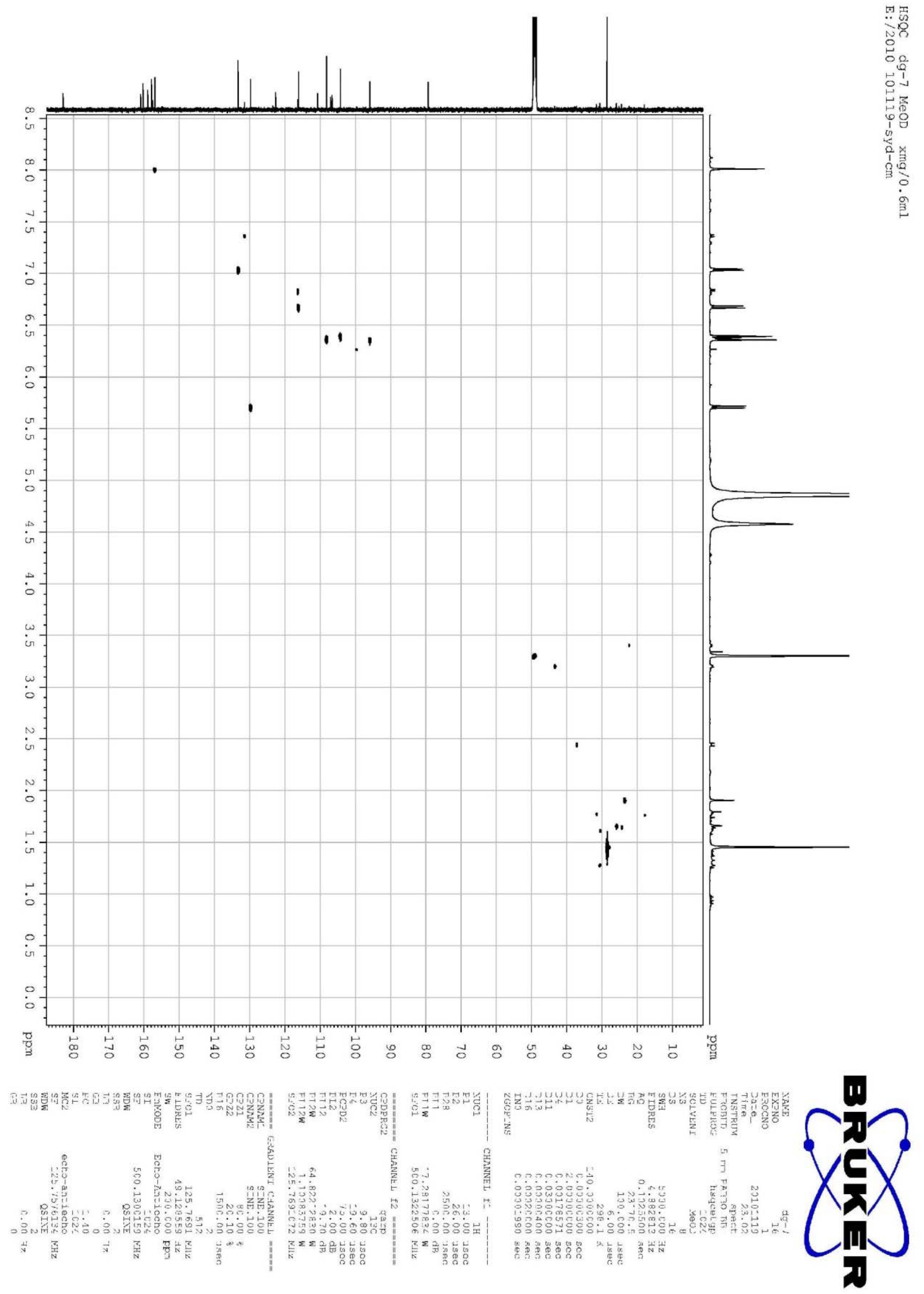

Figure S4. ^{1}H-^{1}H COSY Spectrum of **1** in CD_3OD.

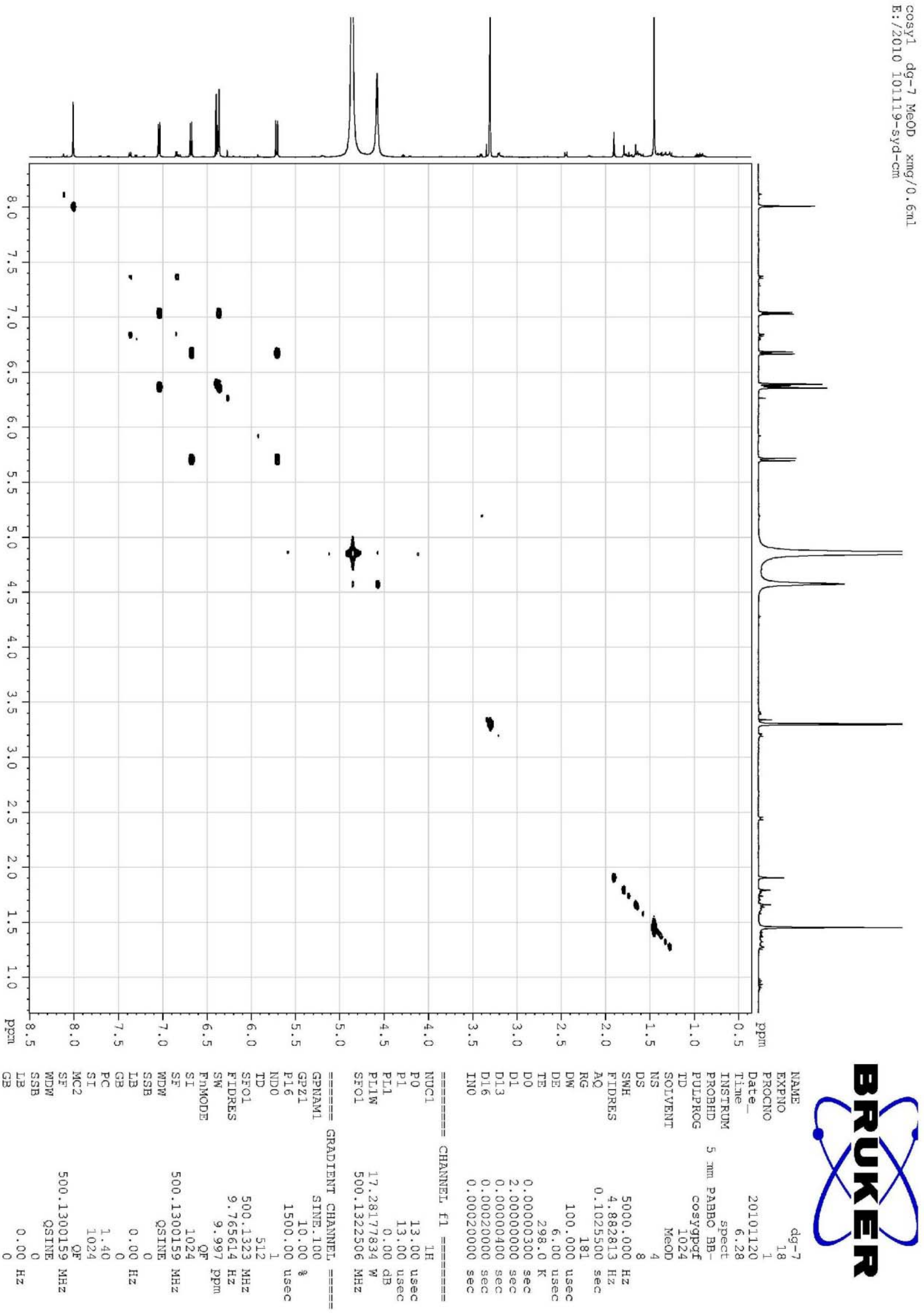

Figure S5. HMBC Spectrum of **1** in CD_3OD.

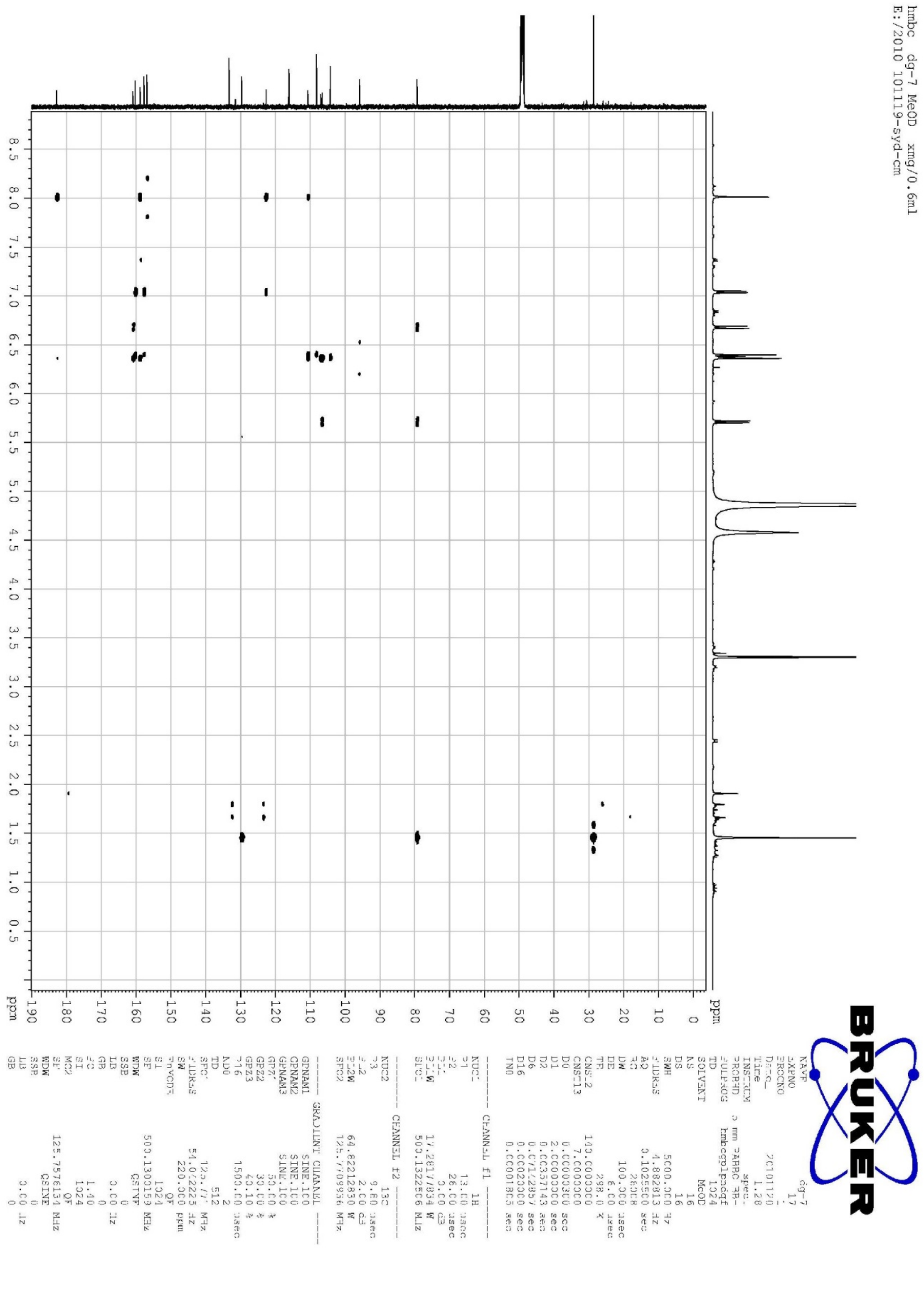

Figure S6. NOESY Spectrum of **1** in CD_3OD.

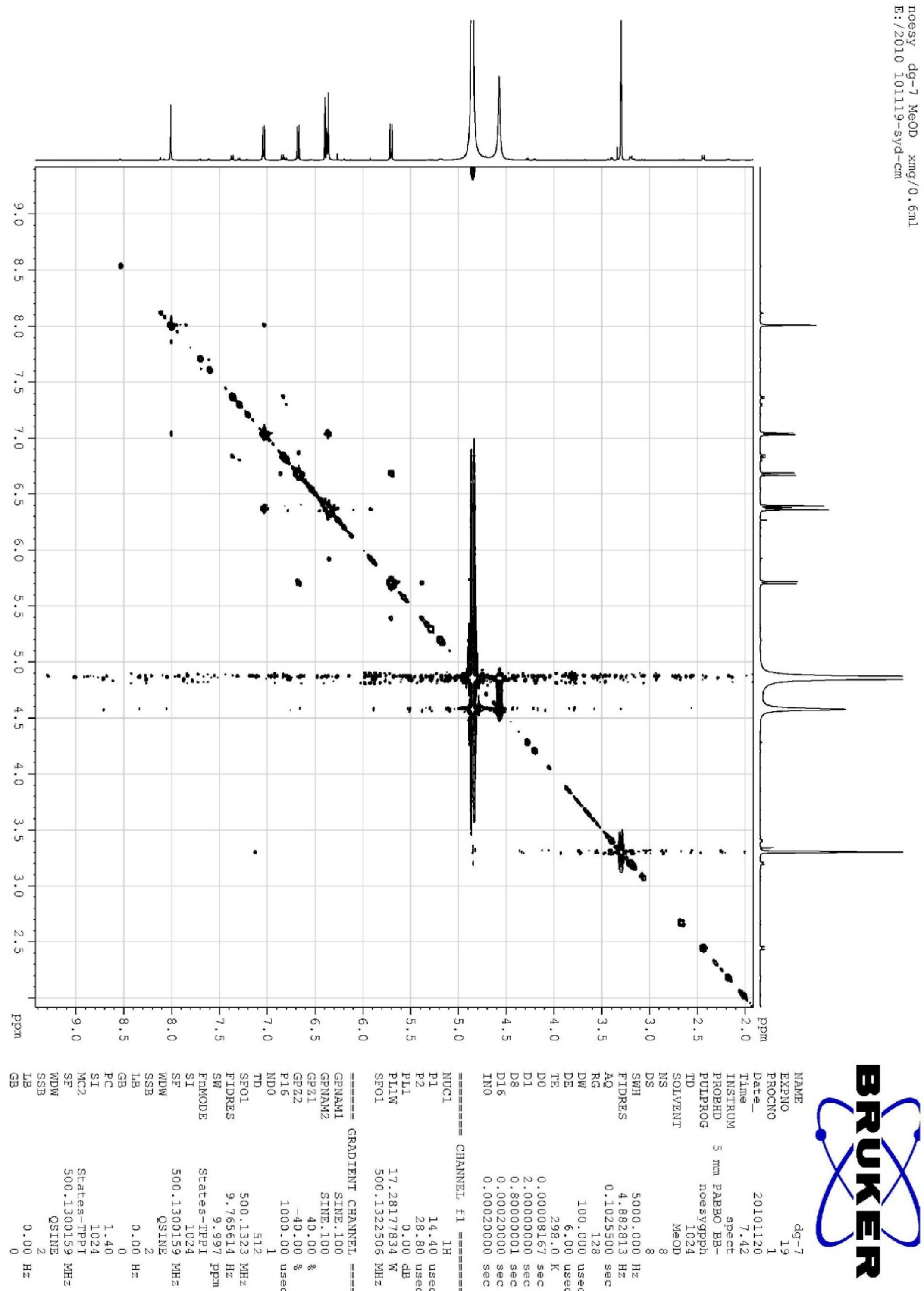

Figure S7. HR-ESI-MS of **1**.

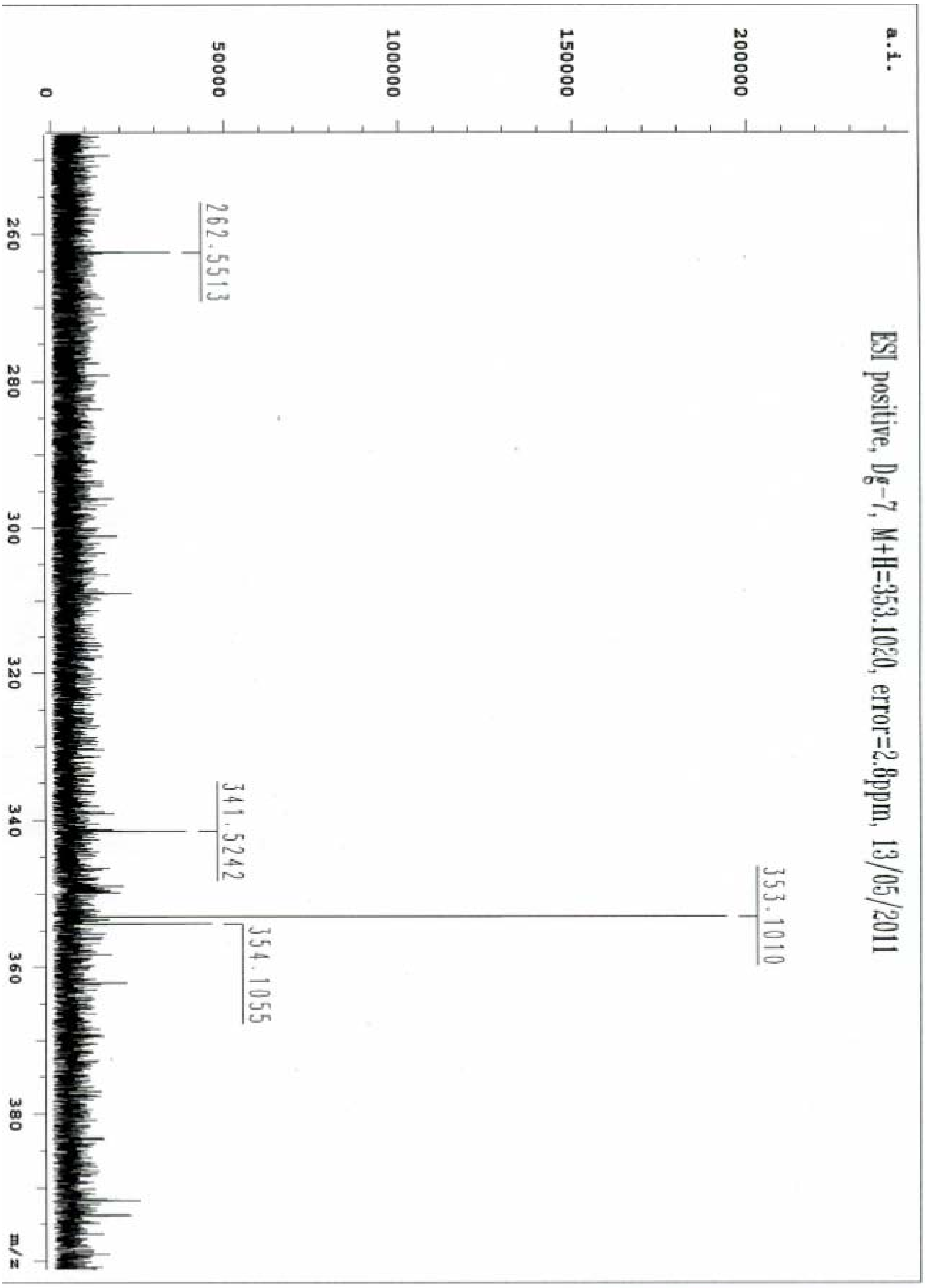

© 2012 by the authors; licensee MDPI, Basel, Switzerland. This article is an open access article distributed under the terms and conditions of the Creative Commons Attribution license (http://creativecommons.org/licenses/by/3.0/).
